# Practical use of transanal decompression tube following the repair of fourth-degree perineal tears associated with vaginal delivery

**DOI:** 10.1186/s40792-024-01966-y

**Published:** 2024-07-05

**Authors:** Hisanori Miki, Kobayashi Toshinori, Hatta Masahiko, Takuki Yagyu, Mitsugu Sekimoto

**Affiliations:** Department of Surgery, Kansai Medical University, 2-3-1 Shinmachi, Hirakata, Osaka 573-1191 Japan

**Keywords:** Fourth-degree perineal tears associated with vaginal delivery, Transanal decompression tube, Anastomotic leakage, Rectovaginal fistula

## Abstract

**Background:**

Fourth-degree perineal tears associated with vaginal delivery (PTAVD) occur in approximately 0.25 to 6% of vaginal deliveries. A persistent challenge in treating fourth-degree PTAVD is the high incidence of anastomotic leakage, leading to impaired quality of life, marked by incontinence, rectovaginal fistula, and painful sexual intercourse. Thus, effective interventions are necessary. Herein, we report our successful approach in repairing a fourth-degree PTAVD, involving the placement of a transanal decompression tube (TDT) during the early postoperative period.

**Case presentation:**

Five patients underwent the repair of fourth-degree PTAVD by suturing the mucosal and muscular layers of the rectum, and the vaginal wall in layers. Subsequently, a TDT was placed in the rectum, positioned 10–15 cm from the anal verge. The TDT was allowed to drain spontaneously without suction. Gastrografin enema examination was performed through a TDT, followed by a computed tomographic scan on postoperative days 3–4. After unfavorable complications were ruled out, the TDT was removed and the patients were transitioned to a normal diet.

**Result:**

All patients showed favorable outcomes with no occurrence of vaginal fistula or incontinence.

**Conclusion:**

This simple intervention demonstrates potential efficacy in reducing anastomotic leakage following the repair of fourth-degree PTAVD.

## Background

The American College of Obstetricians and Gynecologists classifies perineal tears associated with vaginal delivery (PTAVD) into four degrees based on injury severity. Fourth-degree PTAVD involves anal and rectal laceration [1], and occurs in 0.25–6% of vaginal deliveries [2]. Surgical repair is necessary, but a significant challenge is the frequent occurrence of anastomotic leakage [3], leading to complications such as fecal incontinence, painful sexual intercourse, and rectovaginal fistula [1, 4, 5]. Meticulous anatomic approximation during repair of all disrupted layers is recommended to minimize the leakage [5]. However, approximately 25% of patients with third- and fourth-degree PTAVD still experience anastomotic leakage [3]. The challenge of anastomotic leakage is parallel to the challenges of anal-sparing surgery for rectal cancer near the anus, where the incidence reaches to approximately 20%[6]. Severe anastomotic leakage results in lifelong defecation disturbances, such as fecal incontinence. Thus, to prevent anastomotic leakage, the creation of temporary stomas has been prompted. While numerous studies have proven the effectiveness of temporary stomas[7], stomas pose risks such as small bowel obstruction, high output, skin excoriation, herniation, prolapse, and associated surgical closure risks[8, 9]. Therefore, a transanal decompression tube (TDT) is proposed for anastomotic management during rectal surgery [10, 11]. In this study, a TDT was implanted to decompress the anastomosis after repair of 4th degree PTAVD and prevent infection due to stool contamination. The effectiveness of the TDTs was investigated and the results of the initial five cases are reported herein.

## Case presentation

**Fig. 1:**
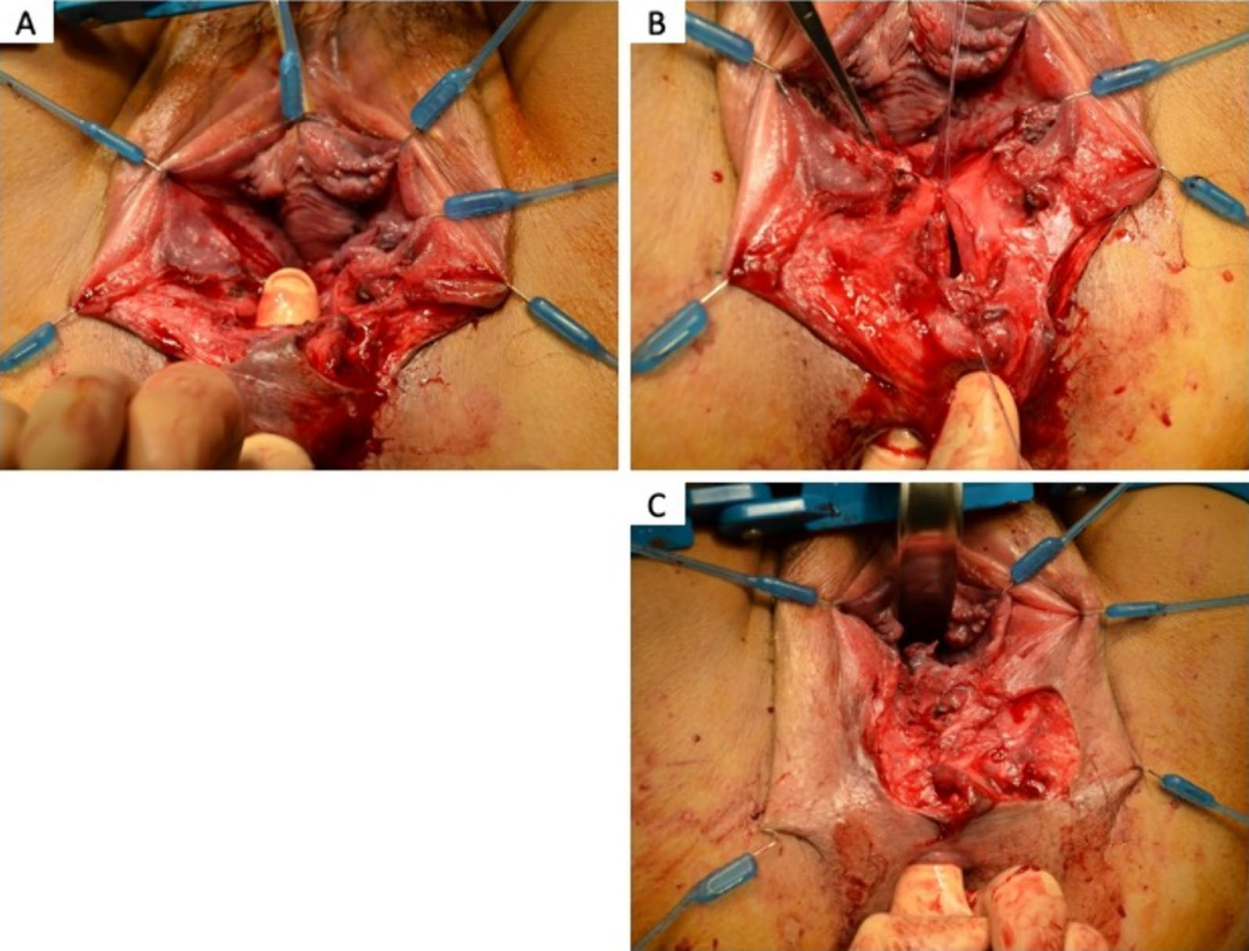
**A** Fourth-degree perineal tears associated with vaginal delivery. The finger passes from anal to vagina. **B** Suture of the rectal mucosal layer with 3-0 absorbable sutures. **C** Suture of the rectal muscular layer with 3-0 absorbable sutures

**Fig. 2:**
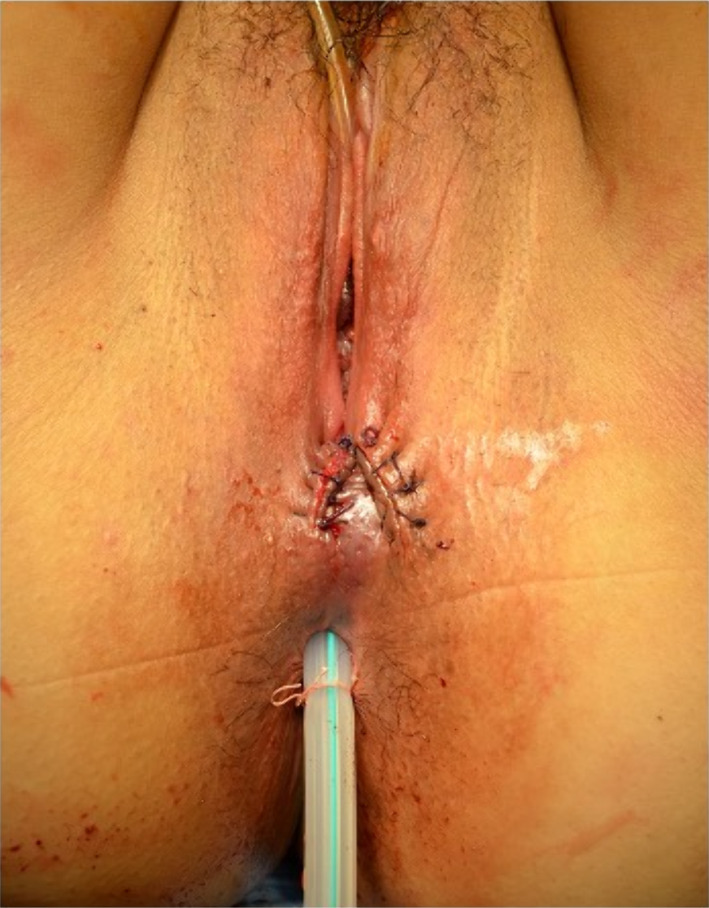
A soft pleated drain was inserted 10–15 cm from the anal verge and secured to the skin using 2-0 silk thread

**Fig. 3:**
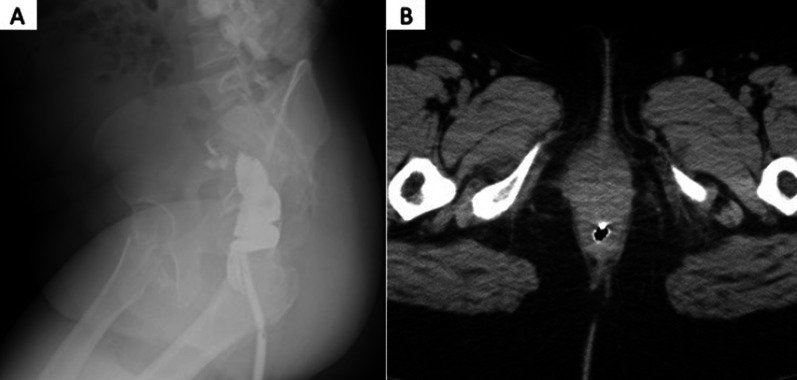
Gastrografin enema (**A**) using a water-soluble contrast medium through the transanal decompression tube, followed by a plain computed tomography (**B**)

Five cases of fourth-degree PTAVD underwent surgical repair in our hospital between January 2019 and May 2022 (Table 1). The median age was 34 years (range: 29–38), median body mass index (BMI) was 21.72 (range: 17.58–36.63), and the median birth weight of the delivered infants was 3345 g (range: 2756–3650 g). All cases involved primiparous individuals, with vacuum-assisted delivery performed in four cases. Postoperatively, all patients received second-generation cephalosporin for 3–5 days. After cleansing the wound, the rectal mucosa and muscular layers were individually closed with 3-0 absorbable sutures. Subsequently, the posterior vaginal wall was closed with 3–0 absorbable sutures (Fig. 1). Finally, a TDT (10 mm soft pleated drain) was placed in the rectum, positioned 10–15 cm from the anal verge (Fig. 2). The TDT was left unaspirated and allowed to excrete spontaneously. Gastrografin, a water-soluble contrast agent was administered through TDT for the enema examination. Immediately after the examination, a plain computed tomographic scan (CT) was performed (Fig. 3) to check for contrast leakage, disruption of wall continuity, and abscesses. After unfavorable complications were ruled out, the patients resumed their normal diet. The median period from operation to gastrografin enema through TDT was 4 days (range: 1–8 days), and the median postoperative hospital stay was 11 days (range: 4–12 days). Case 3, who initially had fourth-degree PTAVD at another hospital, experienced anastomotic leakage seven days postoperatively following the initial suturing. The patient was sent to our hospital for further treatment. Due to the highly contaminated wound, we performed colostomy alongside TDT insertion, considering the high risk of anastomotic leakage despite careful resuturing.

**Table 1:** Patients background

Case	Age (years)	Height (cm)	Weight (kg)	BMI (kg/m^3^)	Reproductive history	Gestational age at birth	Delivery method	Birth weight (g)
1	29	155	88	36.63	G0P0	40w2d	Normal	3045
2	34	159	54.9	21.72	G1P0	40w5d	Vacuum-assisted delivery	3370
3	33	156.7	63.8	25.98	G1P0	41w0d	Vacuum-assisted delivery	3345
4	37	163.5	47	17.58	G1P0	36w2d	Vacuum-assisted delivery	2756
5	38	162	54.8	20.2	G1P0	39w4d	Vacuum-assisted delivery	3650

## Result

In most cases, the TDT drained a minimal amount of stool. All patients demonstrated no evidence of anastomotic leakage on gastrografin enema and CT. Except for Case 3, all patients received laxatives post-TDT extraction. Over a median follow-up period of 2.7 years (range: 1.1–3.9), none of the patients developed rectovaginal fistula or fecal incontinence.

## Discussion

While 0.1–5% of overall PTAVD patients experience anastomotic leakage, the incidence rises significantly to approximately 25% among patients with third- and fourth-degree PTAVD, with 20% facing wound infections within six weeks post-repair surgery [3]. Adequate laceration repair is crucial to prevent rectovaginal fistulas, as around 9% of rectovaginal fistulas in the United States are associated with PTAVD [5]. Postoperative management for third- and fourth-degree PTAVD remains relatively unexplored. Anastomotic leakage is often attributed to wound contamination, infection susceptibility, and unconscious increases in anorectum pressure, frequently disrupting rest at the suture site [12–15]. Intrarectal pressure can rise to 40–60 mmHg, causing involuntary anal contraction [16]. While antibiotics and laxatives are recommended as countermeasures [17], their effectiveness is limited. Therefore, taking more effective measures in preventing anastomotic leakage in fourth-degree PTAVD is essential. A key strategy we developed was to maintain the sutured rectum at rest immediately after suturing. This is considered necessary to prevent anastomotic leakage in anal-sparing surgery for low-lying rectal cancer. Temporary stomas have been utilized and have been proven effective. However, impaired cosmesis and complications, such as intestinal obstruction and skin disorders present as challenges. Furthermore, additional surgery will be required for closure. As a less invasive alternative, TDT offers a promising solution to prevent anastomotic leakage in fourth-degree PTAVD.

The use of TDT as an alternative to diverting stomas was first introduced by Rack et al. in 1966[18]. TDT involves the insertion of a drainage tube through the anus into the rectum to facilitate the expulsion of gas and stool during the early postoperative period. Both temporary stoma and TDT aim to reduce intra-rectal pressure and keep the suture sites at rest. Several studies have compared the effectiveness of temporary stomas and TDT, consistently reporting similar efficacy in reducing the occurrence of anastomotic leakage. [10, 11, 19–24]. However, TDTs may not reduce AL overall in patients undergoing rectal cancer surgery, and they are hardly a replacement for colostomy [22, 25]. Because PTAVD occurs in pregnant women, the disease is not pretreated as it is before rectal cancer surgery, and because of the low anastomosis close to the anus, the usefulness of TDTs may be higher in the treatment of PTAVD than after rectal cancer surgery. However, future case studies are needed.

Previous reports in rectal cancer surgery have suggested TDT indwelling periods ranging from 3 to 7 days, though the optimal duration remains unknown. Complications from TDT are rare; however, intestinal perforation has been reported in cases of long-term indwelling [26]. Early return to normal activities for breastfeeding and childcare is important for patients with perineal lacerations. Therefore, we adopted a strategy of removing the TDT 3 to 4 days after surgery, with the exception of case 3 where failure suture occurred once. Before removal, an enema test using gastrografin and CT was performed to examine for any signs of anastomotic leakage or damage caused by the TDT.

A normal diet was started after the enema test using gastrografin and CT, and no issues were identified in any cases. Once no problems were identified, a normal diet could be started, and long-term outcomes were good. Because adequate nutritional intake is essential for nursing mothers, the contrast method using a TDT suggests the possibility of clarifying when it is safe to start a normal diet.

## Conclusion

We adopted a transanal decompression tube for postoperative management after repair of fourth-degree perineal tears associated with vaginal delivery. The new treatment was safely administered in five cases. Further studies are necessary to confirm their clinical efficacy.

## Data Availability

All the data and materials used in this study were obtained from publicly available sources or databases, and all cited literature is accessible through PubMed.

## Abbreviations

PTAVD: Perineal tears associated with vaginal delivery
CT: Computed tomographic
TDT: Transanal decompression tube
BMI: Body mass index
